# Artificial Intelligence Supports Decision Making during Open-Chest Surgery of Rare Congenital Heart Defects

**DOI:** 10.3390/jcm10225330

**Published:** 2021-11-16

**Authors:** Francesco Paolo Lo Muzio, Giacomo Rozzi, Stefano Rossi, Giovanni Battista Luciani, Ruben Foresti, Aderville Cabassi, Lorenzo Fassina, Michele Miragoli

**Affiliations:** 1 Department of Surgery, Dentistry, Pediatrics and Gynecology, University of Verona, 37134 Verona, Italy; francescopaolo.lomuzio@univr.it (F.P.L.M.); giacomo.rozzi@univr.it (G.R.); giovanni.luciani@univr.it (G.B.L.); 2 Department of Medicine and Surgery, University of Parma, 43126 Parma, Italy; stefano.rossi@unipr.it (S.R.); ruben.foresti@unipr.it (R.F.); aderville.cabassi@unipr.it (A.C.); 3 Humanitas Research Hospital—IRCCS, Via Manzoni 56, 20089 Rozzano, MI, Italy; 4 Department of Electrical, Computer and Biomedical Engineering (DIII), University of Pavia, 27100 Pavia, Italy

**Keywords:** supervised machine learning, right ventricle kinematics, Tetralogy of Fallot, surgery decision making, prognosis prediction

## Abstract

The human right ventricle is barely monitored during open-chest surgery due to the absence of intraoperative imaging techniques capable of elaborating its complex function. Accordingly, artificial intelligence could not be adopted for this specific task. We recently proposed a video-based approach for the real-time evaluation of the epicardial kinematics to support medical decisions. Here, we employed two supervised machine learning algorithms based on our technique to predict the patients’ outcomes before chest closure. Videos of the beating hearts were acquired before and after pulmonary valve replacement in twelve Tetralogy of Fallot patients and recordings were properly labeled as the “unhealthy” and “healthy” classes. We extracted frequency-domain-related features to train different supervised machine learning models and selected their best characteristics via 10-fold cross-validation and optimization processes. Decision surfaces were built to classify two additional patients having good and unfavorable clinical outcomes. The k-nearest neighbors and support vector machine showed the highest prediction accuracy; the patients’ class was identified with a true positive rate ≥95% and the decision surfaces correctly classified the additional patients in the “healthy” (good outcome) or “unhealthy” (unfavorable outcome) classes. We demonstrated that classifiers employed with our video-based technique may aid cardiac surgeons in decision making before chest closure.

## 1. Introduction

Artificial intelligence (AI) has been heralded in the family of “disruptive” technology and as a promising tool to assist clinicians in making better clinical decisions [1,2]. AI implementations can discover and use information hidden in the massive amounts of data usually available for clinical decision making [3,4,5]. Moreover, AI systems aim to reduce diagnostic and therapeutic errors, unavoidable in routine clinical practice, using any sensor or available data to improve the prediction. Among the AI systems that have been tested, there is the Medical Decision Support System which improved clinical decision making in both diagnosis and therapy selection, especially in cases of uncertainty or incomplete information [6].

AI promisingly showed to surpass diagnoses obtained from repetitive human tasks by merging digital imaging with all other data coming from different fields of research [7,8]. Regarding cardiac imaging, AI has been proposed for analyzing CT-scan [9], MRI [10,11] and transthoracic echocardiography data [12].

Recently, AI has been implemented to reduce the number of observations needed to achieve both prognostic and diagnostic robust results [13,14,15,16] with a limited number of patients.

Several AI-related tools, especially for rare disease diagnoses, have shown progress in determining the risk score and in screening areas such as genomics [17], histopathology [18] and radiology [19]. Although all of them are certain to revolutionize our concept of prognosis and diagnosis, AI-related cardiac imaging is currently not employed during open-chest surgery.

Transesophageal echocardiography is the gold standard imaging technique to monitor the left ventricular mechanics during cardiac surgery, but there is still the absence of real-time intraoperative imaging techniques for the evaluation of the right ventricle (RV). This is critical, especially for complex congenital heart diseases in which RV dysfunctions are common features [20]. Hence, AI algorithms are still not employed or developed for this specific task. As for the intraoperative evaluation of the RV, our group has introduced and validated an innovative and contactless imaging technique named Vi.Ki.E. (Video Kinematic Evaluation) [21]. Briefly, the technique consists of recording high-temporal resolution videos of the epicardial movement of the exposed beating heart to calculate kinematic parameters before and after surgery. We applied Vi.Ki.E. in the field of myocardial ischemia [21] and complex congenital heart diseases such as Tetralogy of Fallot (ToF) [22,23], which is the most common RV pathology representing 7–10% of the congenital cardiac defects [24]. Interestingly, in our most recent work on ToF [23], we observed a promising correlation between the before surgery Vi.Ki.E. parameters and preoperative RVEDVI measured by cardiac magnetic resonance (the gold standard for RV evaluation in congenital patients).

In the present work, two supervised machine learning (SML) models were selected and trained with data extracted from twelve consecutive ToF patients undergoing pulmonary valve replacement (PVR). We opted for SML over unsupervised machine learning due to its capability to consider not only the data but also the patient’s documented clinical outcome, reducing the classification error and providing more clinically relevant results [24]. In the operating room, the success and prediction of the outcome of the surgical intervention are still based on the medical team’s experience. Our intraoperative approach allows us to compare the heart kinematic parameters before and after surgery and postulate on the intervention success, but the implementation of SML would allow us to predict the outcome upon chest closure. Therefore, to develop suitable SML models, we extracted features from the frequency domain [25,26,27,28] of the RV epicardial movement recorded with our imaging technique.

This monocentric study aimed to develop Vi.Ki.E.-based SML models to provide an important decision-making tool supporting the medical team during open-chest surgery.

## 2. Materials and Methods

### 2.1. Surgical Methods

The surgical methods and selection criteria of our study were described in our previous work [22]. Briefly, all surgical procedures were performed by one surgeon (G.B.L.) via repeat median sternotomy, using aortic and bicaval cannulation, under normothermic cardiopulmonary bypass on the beating heart. The latter criterion was implemented to exclude the effects of myocardial ischemia/stunning on the kinematic parameters and, thus, the whole recording. Anesthetic was administered only once at the beginning of the surgery. Finally, apart from one patient who had an intraoperative tachycardia that was assessed in our pilot study [21], the heart rate of all patients was the same between the two timepoints.

### 2.2. Right Ventricle Video Recording

**Figure 1:**
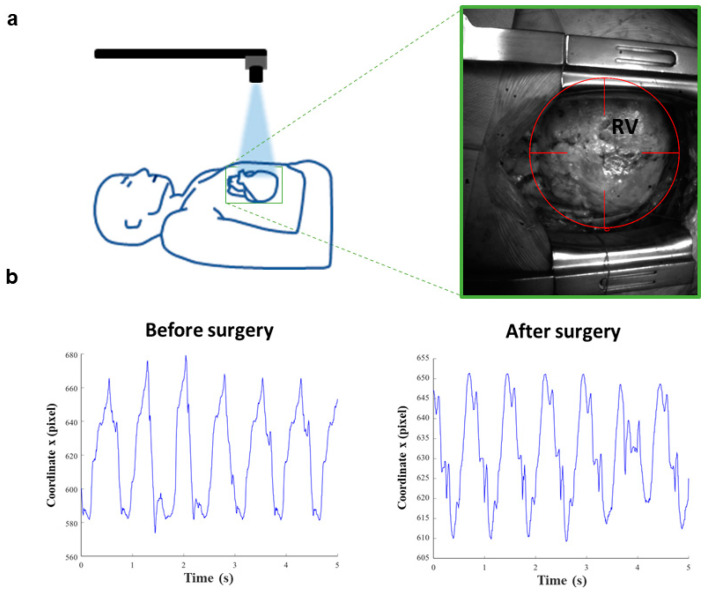
Vi.Ki.E. workflow and coordinates of a representative ToF patient. (**a**) Schematic representation of the Vi.Ki.E. surgical setup (left). The green square surrounding the heart is magnified in the right panel displaying the virtual marker position on the right ventricle (RV) epicardium. (**b**) The x coordinates extracted from a 5 s video recording before and after surgery phases.

The study was approved by the Institutional Review Board (# 847CESC Protocol # 13371) and all patients signed an informed consent agreement. Between November 2016 and November 2018, a total of twelve consecutive ToF patients undergoing PVR were studied accordingly with our previous works [20] and used to train the classifiers. Briefly, three to four videos lasting five seconds were recorded at 200 fps in two surgical phases: before the beginning of the surgical procedure and at the end of the surgery (PVR), circa 30 min after protamine sulphate infusion. The timing of the second surgery was implemented to obtain the same blood density of the before-surgery phase. Consequently, the open source tracking software Video Spot Tracker (VST, CISMM, Computer Integrated Systems for Microscopy and Manipulation, UNC Chapel Hill, NC, USA) was used to track the right ventricle epicardial movement using a virtual video marker (Figure 1a). Then, a custom algorithm implemented in MATLAB^®^ (Release R2020a, The MathWorks, Inc., Natick, MA, USA) extracted the x and y coordinates versus the time of the RV movement (Figure 1b), which were used as a source of information for the SML classifiers.

As concerns the Vi.Ki.E. data, the algorithm provides the following parameters, as described in [21]:

- Maximum contraction velocity: estimates the instantaneous maximal velocity of the cardiac tissue during systole;
- Force: estimates the instantaneous acceleration;
- Energy: estimates the kinetic energy during cardiac cycles;
- Perimeter: estimates the ventricular compliance.

In detail, we have recorded and analyzed a total of 86 videos of beating hearts, subdivided into 43 before and 43 after PVR surgery.

Moreover, we tested our classifiers on two additional patients, with known clinical outcomes and not used for the models’ training, via the decision surface method. One ToF patient undergoing PVR who had a favorable outcome (discharged after 7 days of hospitalization) and one patient undergoing PVR for isolated congenital pulmonary valve regurgitation who had an unfavorable hospital course (death two weeks after surgery).

### 2.3. Features/Predictors

For each video, we extracted numeric features/predictors (Table 1) that are an overall measure of the “trait” of the periodic mechanical x and y coordinates representing the cardiac cycle.

In detail, Table 1 showed the seven MATLAB^®^ functions (Release R2020a, The MathWorks, Inc., Natick, MA, USA) to calculate the features/predictors for each of the 86 video recordings. Furthermore, the known clinical outcome was used to label the patient video recording as unhealthy (before surgery) or healthy (after surgery) class.

### 2.4. Models’ Training and Optimization

The two classifiers showing the highest classification accuracy were trained and optimized with the abovementioned matrix. In detail:

1. Optimizable KNN (k-nearest neighbor classifier), via the “fitcknn” function (https://it.mathworks.com/help/stats/fitcknn.html, accessed on 1 August 2021) with a total of 100 optimization iterations and no standardization of the input features;
2. Optimizable SVM (support vector machine classifier), via the “fitcsvm” function (https://it.mathworks.com/help/stats/fitcsvm.html, accessed on 1 August 2021) with a total of 100 optimization iterations and no standardization of the input features.

Both models were optimized via the minimization of the classification error and, also, 10-fold cross-validated: for 10 times, a new 10% of the patients were not used to train but to blindly validate the models. Cross-validation is a good practice to avoid over-fitting, which is undesired memorization of the training data reducing the model predictive ability.

### 2.5. Decision Surface

The cross-validated and optimized classifiers were used to build 2D simplified classification models for both x and y coordinates. This simplified 2D approach allowed to build the so-called decision surface where every point of the Cartesian plane is classified using, as a representative feature of the patient status, the SNR of the kinematic periodic movement of the RV.

### 2.6. Statistical Analysis

To calculate the minimum number of patients to perform the study, we used the formula by Armitage et al. [29] to evaluate the 95% confidence interval for the disease’s prevalence, where the amplitude (D) of that confidence interval is established a priori and with Z(_σ⁄2_) = 1.96. Knowing the ToF prevalence is circa 1/3500 live births and establishing D = 2%, the minimum number of patients that should be enrolled to study the disease is N > 10.97 [30].

The Vi.Ki.E. parameters are expressed as mean ± SEM. The normality of the distribution of the data was investigated with Kolmogorov–Smirnov test, and the significance was assessed by Mann–Whitney test. *p* values were considered significant at <0.05.

## 3. Results

### 3.1. Selected Models

The frequency-domain-related features extracted from the epicardial movement of the RV were processed by the Classification Learner Application in MATLAB^®^ to test different classification models.

Table 2 shows that the two ensemble methods, as well as the linear discriminant, the kernel naïve Bayes and the Gaussian naïve Bayes models, displayed a classification accuracy below 75% and therefore were not adopted in this work [31].

On the contrary, both KNN and SVM classifiers showed the highest classification accuracy and therefore were elected to be customized and optimized for our specific medical aim.

### 3.2. Optimized Model Training

**Figure 2:**
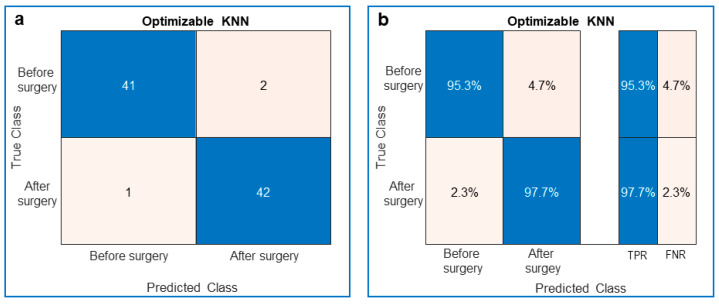
Performance of the trained optimizable k-nearest neighbor (KNN) classifier. (**a**) Confusion matrix showing the true and predicted classes of the ToF patients’ videos. The blue diagonal is related to the number of videos that were truly recognized (Predicted Class = True Class), whereas the pink antidiagonal is related to the number of videos that were falsely recognized (Predicted Class ≠ True Class). (**b**) Same as (**a**) with the True Positive Rate (TPR) in blue and the False Negative Rate (FNR) in pink.

**Figure 3:**
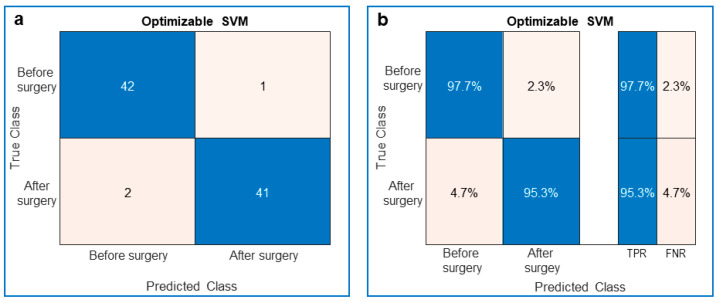
Performance of the trained optimizable support vector machine (SVM) classifier. (**a**) Confusion matrix showing the true and predicted classes of the ToF patients’ videos. The blue diagonal is related to the number of videos that were truly recognized (Predicted Class = True Class), whereas the pink antidiagonal is related to the number of videos that were falsely recognized (Predicted Class ≠ True Class). (**b**) Same as (**a**) the True Positive Rate (TPR) in blue and the False Negative Rate (FNR) in pink.

**Figure 4:**
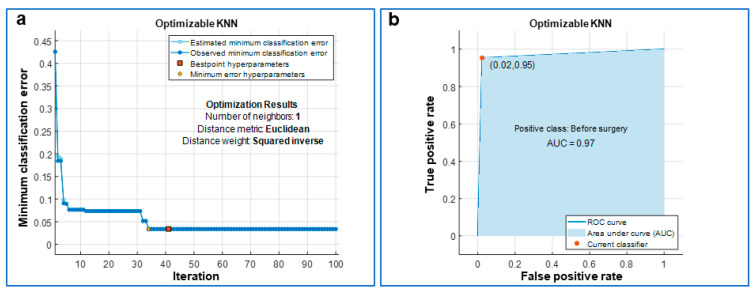
Optimization of the k-nearest neighbor (KNN) classifier. (**a**) The classifier was optimized over 100 iterations via the minimization of the classification error and its optimized hyperparameters are reported as “Optimization Results”. In detail, for each iteration of the optimization, the classifier was also 10-fold cross-validated. (**b**) Receiver Operating Characteristic (ROC) curve with the area under the curve (AUC) painted in blue; a value of AUC close to 1 means a very low classification error for the optimized classifier.

**Figure 5:**
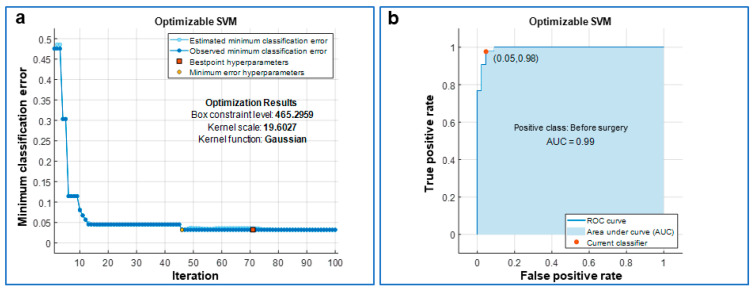
Optimization of the support vector machine (SVM) classifier. (**a**) The classifier was optimized over 100 iterations via the minimization of the classification error and its optimized hyperparameters are reported as “Optimization Results”. In detail, for each iteration of the optimization, the classifier was also 10-fold cross-validated. (**b**) Receiver Operating Characteristic (ROC) curve with the area under the curve (AUC) painted in blue; a value of AUC close to 1 means a very low classification error for the optimized classifier.

At the end of the optimized training we obtained:

- The confusion matrix with the number of truly recognized videos on the diagonal and the number of falsely recognized videos on the antidiagonal (Figure 2a and Figure 3a);
- The preceding confusion matrices were used to compute the corresponding True Positive Rate (TPR), the probability to truly recognize the class, on the diagonal, and False Negative Rate (FNR), the probability to misrecognize the class, on the antidiagonal (Figure 2b and Figure 3b);
- The optimized parameters of the 10-fold cross-validated models (Figure 4a and Figure 5a);
- The Area Under Curve (AUC) or area under ROC (Receiver Operating Characteristic) curve (Figure 4b and Figure 5b);
- The MATLAB^®^’s script of the optimized model;
- The MATLAB^®^’s optimized model (as a saved workspace structure array) to employ in the operating room to classify the current patient’s heart movement as unhealthy or healthy.

In detail, the KNN displayed a TPR of 95.3% and an FNR of 4.7% before surgery, whereas after surgery the TPR was 97.7% and the FNR was 2.3% (Figure 2).

Similarly, the SVM showed a TPR of 97.7% and an FNR of 2.3% before surgery, whereas after surgery the TPR was 95.3% and the FNR was 4.7% (Figure 3). When the cross-validated classifier reached the last optimization iteration (the 100th in our study), each iteration was evaluated in terms of classification error and the one with the minimum error provided the so-called “best point hyperparameters”. The estimated and observed minimum classification errors were plotted against the iteration number when they were <0.05. The KNN was optimized for a number of neighbors equal to 1, for the Euclidean distance metric and the squared inverse distance weight (Figure 4). The SVM was optimized considering a Gaussian kernel function, a kernel scale of 19.6027 and a box constraint level of 465.2959 (Figure 5). In addition, for both optimized models, the AUC was close to 1 (0.97 for KNN and 0.99 for SVM), which is the value of a perfect classification (Figure 4 and Figure 5).

### 3.3. Classifiers’ Prediction Ability Tested via Two Additional Patients with Different Outcomes

**Figure 6:**
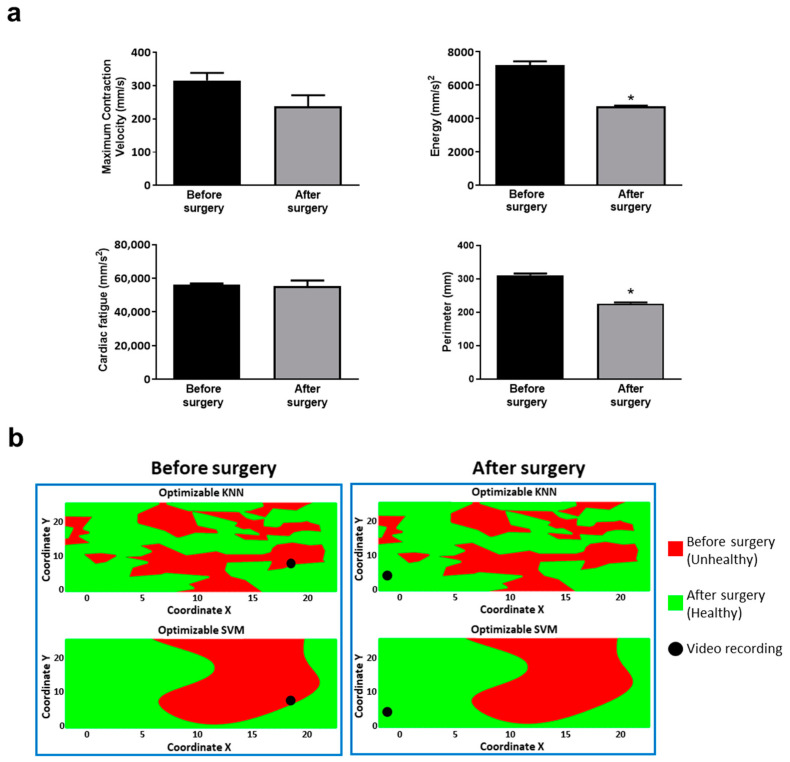
Vi.Ki.E. data and decision surfaces of a ToF patient with a favorable outcome. (**a**) The average Vi.Ki.E. parameters calculated before (black) and after surgery (grey). Top left: Maximum contraction velocity. Top right: energy. Bottom left: force. Bottom right: perimeter. Data are shown as mean ± SEM. * *p* < 0.05 versus before surgery. (**b**) The decision surfaces of the signal-to-noise ratio (SNR) for both our classification models before (left panels) and after surgery (right panels). The black circle represents the classification/prediction of the patient’s video according to KNN (top) and SVM (bottom) models. The red area is related to a prediction of unhealthy, whereas the green area to a prediction of healthy.

We sought to investigate the classifiers’ prediction skills in two additional patients not considered in the training process. First, we assessed the cardiac kinematics (Figure 6a) in one ToF patient who underwent PVR with a known favorable outcome (discharged after 7 days of hospitalization). As in our previous works [22,23], we observed a decreasing trend for all Vi.Ki.E. parameters, which was significant for the energy and perimeter. Then, the Vi.Ki.E. coordinates of this patient were inputted into our SML models to build the decision surfaces. As a representative feature, we selected the signal-to-noise ratio (dB) (SNR) displayed in Figure 6b. Both before and after surgery, video recordings were correctly classified into the unhealthy and healthy classes by both trained models, respectively.

**Figure 7:**
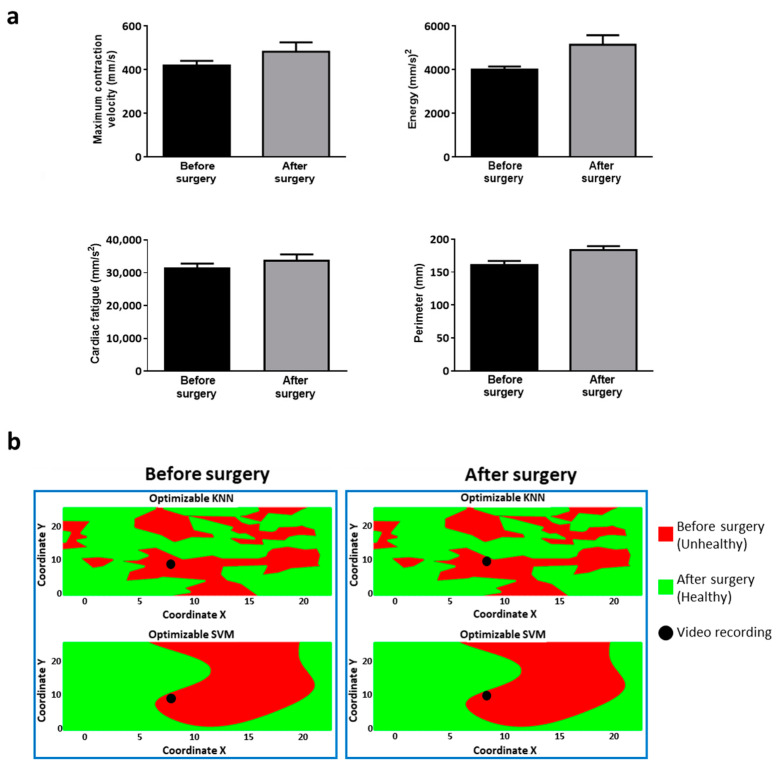
Vi.Ki.E. data and decision surfaces of a patient with pathophysiology similar to ToF and an unfavorable outcome. (**a**) The Vi.Ki.E. parameters calculated for every recording before (black) and after surgery (grey). Top left: Maximum contraction velocity. Top right: energy. Bottom left: force. Bottom right: perimeter. Data are shown as mean ± SEM. (**b**) The decision surfaces of the signal-to-noise ratio (SNR) for both our classification models before (left panel) and after surgery (right panel). The black circle represents the classification/prediction of the patient’s video according to KNN (top) and SVM (bottom) models. The red area is related to a prediction of unhealthy, whereas the green area is related to a prediction of being healthy.

Likewise, we assessed the cardiac kinematics (Figure 7a) in a patient undergoing PVR for isolated congenital pulmonary valve regurgitation who had an unfavorable hospital course (death two weeks after surgery). We observed an increasing trend for all the Vi.Ki.E. parameters, which is in contrast with our previous work. The Vi.Ki.E. coordinates of this patient were used to build the decision surfaces for the SNR feature (Figure 7b). In detail, the before-surgery video recordings were correctly classified into the unhealthy class by both trained models (Figure 7: left panels, top and bottom). On the contrary, the after-surgery video recordings were still classified into the unhealthy class (instead of healthy) by both trained models (Figure 7: right panels, top and bottom), thus correctly predicting the documented unfavorable outcome.

## 4. Discussion

In this work, we employed supervised classifiers capable of correctly predicting outcomes on a set of patients affected by ToF and undergoing PVR. Tetralogy of Fallot is a rare congenital heart disease, which requires repair in early infancy and is associated with excellent survival into adulthood. Later on, most patients will develop chronic pulmonary valve regurgitation, which is characterized by the impairment of the right ventricular mechanical function [39]. The lack of imaging techniques for the intraoperative evaluation of the RV does not allow a precise assessment of its mechanical function during surgery, comparable to the information provided by MRI outside the operating room setting [40]. Moreover, the kinematics of the RV are extremely complex and there are emblematic cases in which its movement could be very atypical [41].

Predictive outcomes based on conventional technologies require a vast dataset of patients; conditions that, for rare diseases, entail multi-center investigations [42]. To overcome this limitation, a promising tool could be SML classifiers due to their ability to merge data coming from different research fields.

In this study, we trained two SML classifiers based on Vi.Ki.E., a contactless imaging technology developed by our laboratories to evaluate the RV kinematics [21,22,23,43]. We used the Vi.Ki.E. coordinates to extract frequency-domain-related features/predictors from the sinusoidal-like movement of the heart. The outputs of the trained classifiers are two classes, named either unhealthy (before surgery) or healthy (after surgery). The two models with the highest classification accuracy were the KNN and the SVM (>75%), which were optimized for our specific medical aim. Interestingly, both KNN and SVM classifiers are widely used in cardiology/cardiac surgery. For example, KNN has been recently employed for the accurate delineation of the QRS complex [44] and as predictive analytics for the postoperative length of stay after isolated coronary artery bypass grafting [45]. Likewise, SVM has been extensively used for predicting the development of complications after cardiac surgery [46,47]. Indeed, Moghaddasi et al. employed the SVM model to classify the severity of mitral regurgitation via transthoracic echocardiography [48].

Despite the limited number of cases, as shown in the power analysis consistent with the rarity of ToF, we optimized and 10-fold cross-validated the SMLs, obtaining good results in terms of TPR, FNR and AUC for both KNN and SVM models.

The usefulness of both classifiers was tested with an additional ToF patient undergoing PVR who had a favorable outcome. The Vi.Ki.E. assessment showed a decreasing trend for all kinematic parameters in line with our previous studies on right ventricular unloading [22,23]. We used the signal-to-noise ratio (SNR) predictor as a representative feature to build a simplified 2D classification model, named “decision surface”, for both coordinates. Both KNN and SVM decision surfaces correctly recognized the patient’s before and after surgery status in the respective unhealthy and healthy classes, which is thus in line with his prognosis.

We also used our classifiers on another patient who died two weeks after PVR for isolated congenital pulmonary valve regurgitation. The intraoperative Vi.Ki.E. parameters showed the opposite trend to that of our ToF population, denoting a still fatigued heart, although supported by inotropic drugs. As shown by the decision surfaces, the two classification models classified the after surgery heart movement as unhealthy (instead of healthy), thus correctly highlighting the still complex clinical condition. In conclusion, both KNN and SVM models classified the dead patient in line with his adverse prognosis.

The intraoperative clinical situation of the patient with an unfavorable outcome demonstrated the necessity of a real-time classification tool, directly applicable in the operating room [49], that could be useful to surgeons for prognostic assessment [50]. It is conceivable that the patient who did not benefit from PVR had since developed irreversible right ventricular dysfunction, due to longstanding RV hypertrophy and dilation, ultimately resulting in myocardial scarring (replacement of muscle with connective tissue). The quantification of scar tissue deposition relies on preoperative cardiac MRI. However, the extent of myocardial scarring, which may predict irreversible right ventricular dysfunction, is presently unclear. Therefore, any additional tool which may aid in predicting postoperative recovery, including intraoperative technology integrated with AI elaboration, is potentially applicable and beneficial.

This study aimed to create ready-to-use classifiers to support surgeons during open-chest surgery. As both Vi.Ki.E. and classifiers’ algorithms were implemented in MATLAB^®^, the SMLs can be employed within the operating room right after the video-kinematic evaluation, providing insights for the intraoperative prognosis. Another strength of our classifiers is their customizable and optimizable nature, given that the proposed framework could be applied in other cardiac pathological conditions after training with specific patients’ datasets. Moreover, the models can be improved over time as new patients are followed up and included in the training dataset. Our single-center pilot study demonstrated that, in the context of rare congenital heart diseases, predictive classification can be achieved with a minimal number of patients by resorting to artificial intelligence tools.

## Figures and Tables

**Table 1:** Set of features/predictors extracted from the periodic movement of the RV. A total of 7 features/predictors are calculated for both x and y coordinates. A citation for each feature is shown in the table (each link was accessed on 1 August 2021).

Feature/Predictor	MATLAB^®^ Function
Band power [pixel]: it returns the ‘average power’ or average *l*_2_ norm (average Euclidean norm) of the input signal [pixel]	bandpower https://it.mathworks.com/help/signal/ref/bandpower.html
Power bandwidth [Hz]: it returns the 3 dB (half-power) bandwidth of the input signal	powerbw https://it.mathworks.com/help/signal/ref/powerbw.html
Occupied bandwidth [Hz]: it returns the 99% occupied bandwidth of the input signal	obw https://it.mathworks.com/help/signal/ref/obw.html
Spurious free dynamic range [dB]: it returns the SFDR of the real sinusoidal-like input signal	sfdr https://it.mathworks.com/help/signal/ref/sfdr.html
Signal to noise and distortion ratio [dB]: it returns the SINAD of the real sinusoidal-like input signal	sinad https://it.mathworks.com/help/signal/ref/sinad.html
Signal to noise ratio [dB]: it returns the SNR of the input signal	SNR https://it.mathworks.com/help/signal/ref/snr.html
Spectral entropy (information content) of the input signal	pentropy https://it.mathworks.com/help/signal/ref/pentropy.html

**Table 2:** All tested classifiers ranked in terms of accuracy (%).

Classifier	Accuracy (%)
Boosted trees (ensemble of trees using the AdaBoost (Adaptive Boosting) algorithm) [32]	46.5
RUSBoosted trees (ensemble of trees using the RUSBoost (Random Undersampling Boosting) algorithm) [33]	46.5
Linear discriminant [34]	66.3
Kernel naïve Bayes [35]	68.6
Gaussian naïve Bayes [36]	69.8
Fine Gaussian (Radial Basis) support vector machine (SVM) [37]	79.1
Fine *k*-nearest neighbor (KNN) [38]	86.0

## Data Availability

Data will be provided upon request.
